# Supplementary material for: Machine-learning-assisted and real-time-feedback-controlled growth of InAs/GaAs quantum dots
Source: Nat Commun. 2024 Mar 29;15:2724. doi: 10.1038/s41467-024-47087-w (PMC10980817; doi:10.1038/s41467-024-47087-w)
Supplement: Supplementary file 1 — Supplementary Information [file 41467_2024_47087_MOESM1_ESM.pdf]

Supplementary Information

Machine-Learning-Assisted and Real-Time-Feedback-Controlled Growth of InAs/GaAs Quantum Dots

Chao Shen[1,2,3,#], Wenkang Zhan[1,2,#], Kaiyao Xin[2,4], Manyang Li[1,2], Zhenyu Sun[1,2], Hui Cong[2,5], Chi Xu[2,5], Jian Tang[6], Zhaofeng Wu[3], Bo Xu[1,2], Zhongming Wei[2,4], Chunlai Xue[2,5], Chao Zhao[1,2,*], and Zhanguo Wang[1,2]

[1] Laboratory of Solid State Optoelectronics Information Technology, Institute of Semiconductors, Chinese Academy of Sciences, Beijing 100083, China

[2] College of Materials Science and Opto-Electronic Technology, University of Chinese Academy of Science, Beijing 101804, China

[3] School of Physics Science and Technology, Xinjiang University, Urumqi, Xinjiang 830046, China

[4] State Key Laboratory of Superlattices and Microstructures, Institute of Semiconductors, Chinese Academy of Sciences, Beijing 100083, China

[5] Key Laboratory of Optoelectronic Materials and Devices, Institute of Semiconductors, Chinese Academy of Sciences, Beijing 100083, China

[6] School of Physical and Electronic Engineering, Yancheng Teachers University, Yancheng 224002, China

*Email: zhaochao@semi.ac.cn

#Equally contributing authors

1. RHEED characteristics of QDs with different labels

Time series of reflection high-energy electron diffraction (RHEED) images at the beginning stages of growth were presented in Figure S1. The RHEED patterns of GaAs substrates were predominantly observed as streaks, as shown in Figure S1a. Even though a small amount of InAs is deposited when the In shutter is opened, the RHEED images still exhibited prominent streak patterns as shown in Figure S1b. Therefore, it is challenging to accurately label different growth states at the initial stages of material growth.

As the growth going, the streaks in the RHEED pattern were significantly less clear than those of GaAs when the QD density was zero, as shown in Figure S1c. This can be attributed to the fact that the adhesion of In atoms on the substrate is weak, particularly at higher substrate temperatures, making it easy for them to diffuse or migrate. As a result, it becomes difficult for the material surface to form an ordered structure. Consequently, the streaks in the RHEED pattern are not as clear as the in the GaAs ordered structure.[1]

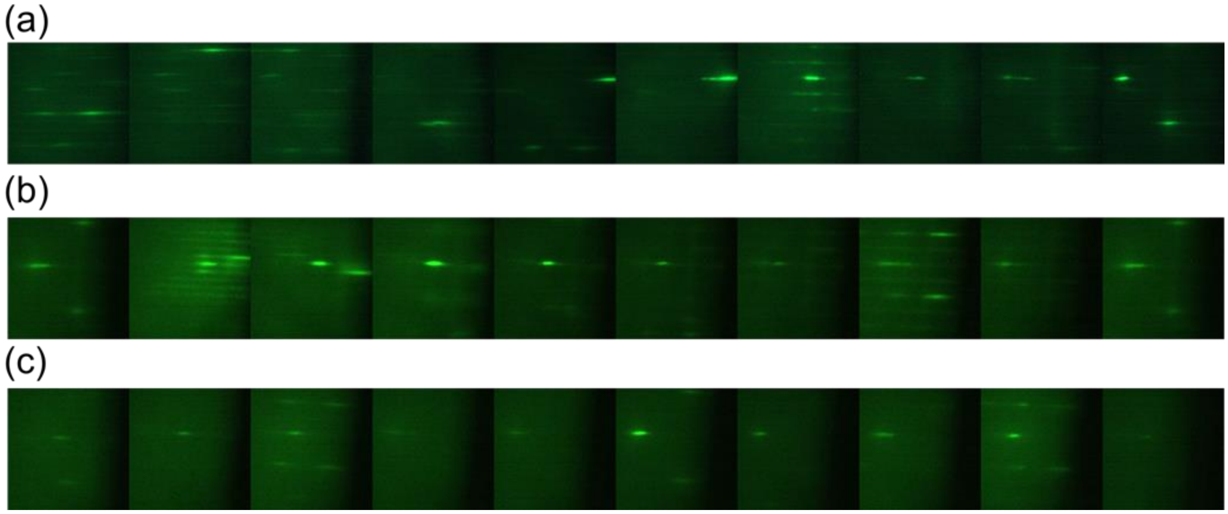

Figure S1. Ten typical reflection high-energy electron diffraction (RHEED) images collected continuously. (a) GaAs substrates before growth started, (b) after the In shutter opened for a few seconds, and (c) samples with zero-density QDs.

When comparing RHEED images with different labels before the QD formation, it is difficult to distinguish differences. As shown in Figures S2a, S2b, and S2c, these patterns exhibit features resembling those from zero-density QD samples. When a RHEED pattern appears during growth that matches an existing pattern in the database, it can be used as a prediction of the outcome of the current growth conditions. Therefore, obtaining a dataset that collects RHEED patterns with varying parameters as comprehensive as possible is crucial. Otherwise, the model may struggle to accurately classify the information during deployment.

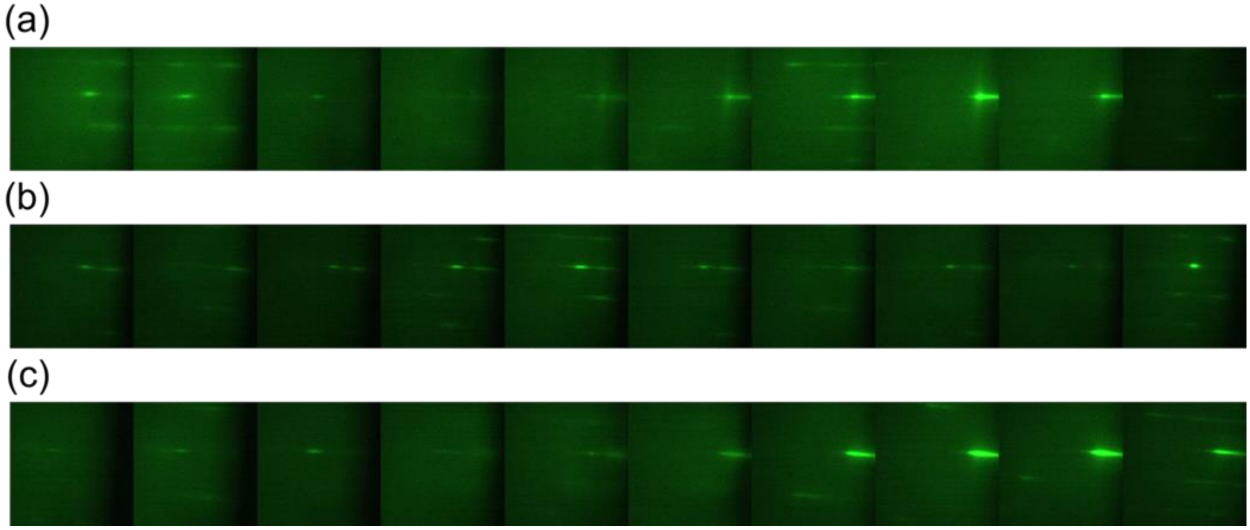

Figure S2. Ten consecutive reflection high-energy electron diffraction (RHEED) images with different labels obtained before the quantum dot (QD) formation. (a) “low” label, (b) “middle” label, and (c) “high” label.

When comparing RHEED patterns after the QD formation, those with “low” labels consistently exhibit a superposition of streaks and spots, as shown in Figures S3a. The streaks correspond to a pattern with the InAs wetting layer information.[2] In contrast, the smaller spots correspond to QDs formed by stress relaxation.[3, 4] These streaks and spots overlap and appear repeatedly in a periodically varying RHEED sequence, as illustrated in Figure S2b. Moreover, the shape of the specular spot gradually changes after the QD formation. As QD density increases, the specular spot gradually became more intense, as shown in Figures S3b and S3c.[5]

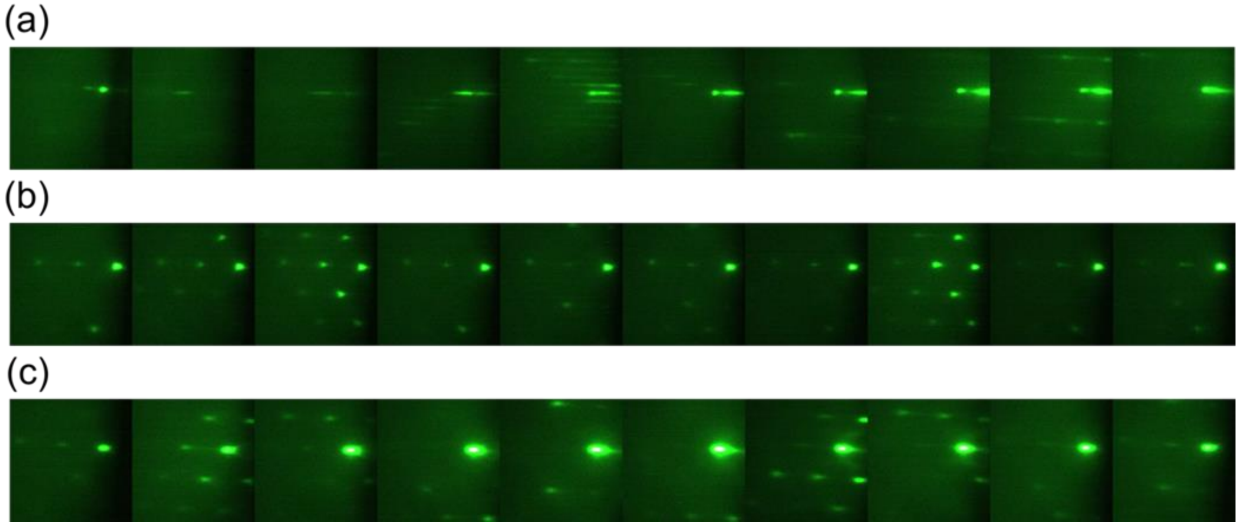

Figure S3. Ten consecutive reflection high-energy electron diffraction (RHEED) images with different labels obtained after the quantum dot (QD) formation. (a) “low” label, (b) “middle” label, and (c) “high” label.

2. The cropping area of RHEED images

Before inputting data into the model, it is necessary to select appropriate regions from the original RHEED images. It is crucial to ensure that the cropped images contain as much relevant information as possible while avoiding capturing too much of the RHEED fluorescent screen window area.[6] Thus, we adopted the approach shown in Figure S4. The cropping box should encompass the specular spot and complete information of the two first-order streaks and at least one second-order streak.[7] Additionally, the edge of the selection box should have a certain distance from the nearest streak.

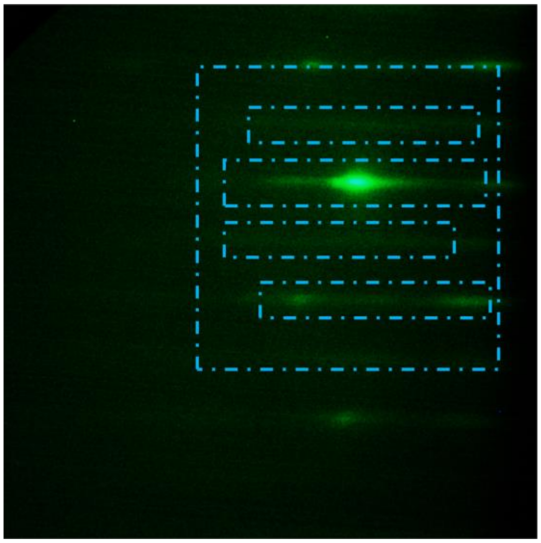

Figure S4. A schematic of the image cropping. The largest box indicates the extent of the cropped areas, and within the largest box, from top to bottom, the first-order streak, the specular spot, another first-order streak, and the second-order streak.

3. The number of images selected

We used 10% of the dataset for training to study the impact of the number of images on model accuracy. After training for the same epoch in a 3D ResNet 50 model, it can be observed that as the number increases, the validation accuracy of both the QDs model and density model gradually improves, as shown in Figure S5. However, when the number of selected images is greater than 8, the accuracy increase is no longer evident. So, choosing 8 images as a sample is reasonable to avoid excessive redundancy of model parameters.

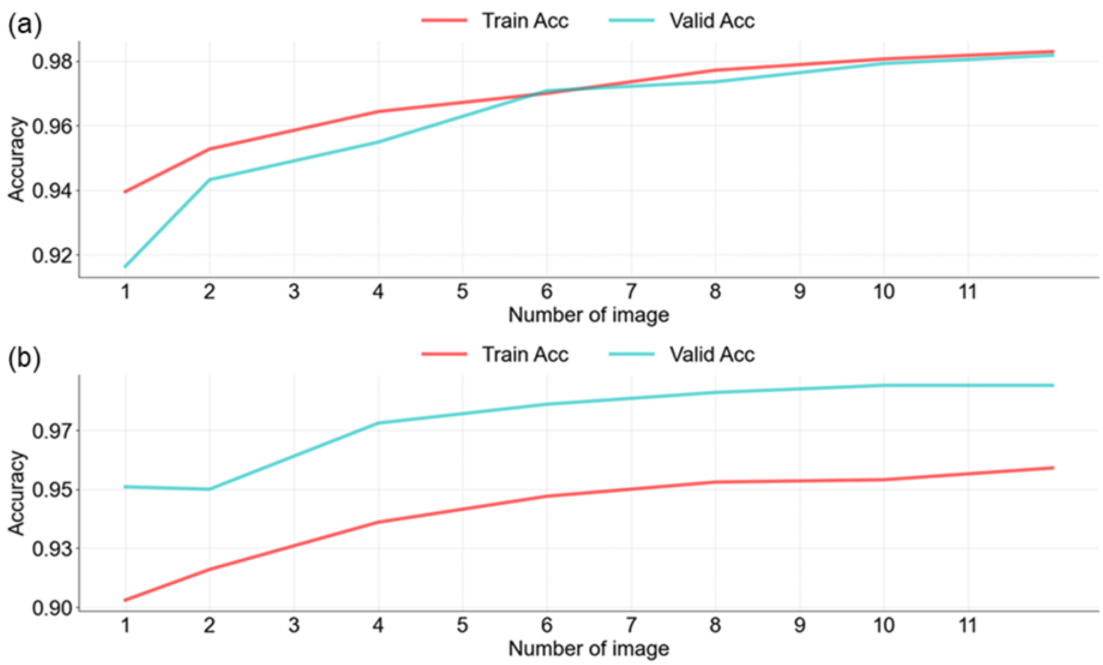

Figure S5. The number of images selected. (a) The QDs model and (b) the density model train and valid accuracy with different numbers of images. Source data are provided as a Source Data file.

4. Speed management

It is essential to conduct data preprocessing operations before initiating the model training. This approach serves two purposes: firstly, it reduces the time and memory consumption associated with data augmentation and preprocessing during the training process, and secondly, it allows the currently trained model to run in parallel with the data preprocessing of other models. As shown in Figure S6, if the data is processed during training, as the number of images to be processed increases, the model data processing speed significantly decreases. More precisely, the training speed decreased from 89 to 14 samples per second, and the validation speed dropped from 136 to 15 samples per second. On the contrary, if the data has been preprocessed and converted into a

NumPy array, which only needs to be quickly called during the training process, the data processing speed will not be significantly slow when a single NumPy array becomes larger.

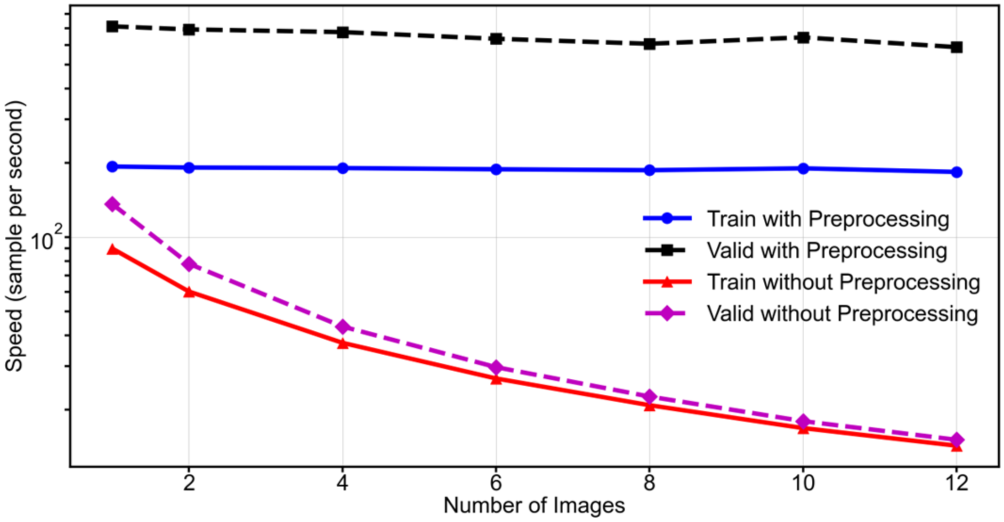

Figure S6. Speed management. Model training and validation speed before and after preprocessing with different numbers of images. Source data are provided as a Source Data file.

5. The detailed structure of the ResNet 50 model

Our 3D ResNet 50 model employs 3D convolutions and 3D batch normalization. The convolutional kernel size is 3 × 3 × 3, with a stride of 1 and padding of 1. Additionally, the essence of the 3D ResNet 50 lies in its residual blocks within the model we employed. Two basic residual blocks residual blocks are utilized: Residual Block 1 and Residual Block 2, as depicted in Figures S7a and S7b, respectively. Figure S7a illustrates two pathways for data processing, one utilizing identity shortcuts and the other combining two sets of 3D convolutional layers with 3D batch normalization connected via the rectified linear unit (ReLU) activation function. Using identity shortcuts enables specific input data to pass through layers directly, preventing information loss and addressing gradient vanishing, reducing computational load by bypassing convolutional layers and batch normalization, leading to efficient training and model size maintenance.[8, 9] Figure S7b

illustrates a residual structure where the path utilizing identity shortcuts in Figure S7a has been replaced by 3D convolutional kernels with a size of 3 × 3 × 3, a stride of 1, and padding of 2, along with 3D batch normalization. In the other pathway, 3D convolutional kernels retain the size of 3 × 3 × 3.

Nevertheless, there are variations in the stride and padding configurations. The first 3D convolutional layer has a stride of 1 and padding of 1, whereas the second 3D convolutional layer has a stride of 2 and padding of 1. Compared to the original ResNet 50 with a stride of 2, causing about 75% information loss, beginning the 3D ResNet 50 setup with a stride-1 3D convolutional layer is beneficial, effectively limiting information loss during downsampling.[10, 11] So, basic residual blocks promptly drop unfavorable training parameters, thereby efficiently train deep neural networks and simultaneously learn temporal and spatial relationships within video data.[12, 13, 14] Based on two basic residual blocks, other residual structures in the model were composed, namely, Basic Block 1, Basic Block 2, Basic Block 3, and Basic Block 4, respectively. Each residual block is constructed by stacking multiple convolution layers and basic residual blocks. Before these residual structures are connected, there is also a Basic Stem, as shown in Figure S7c.

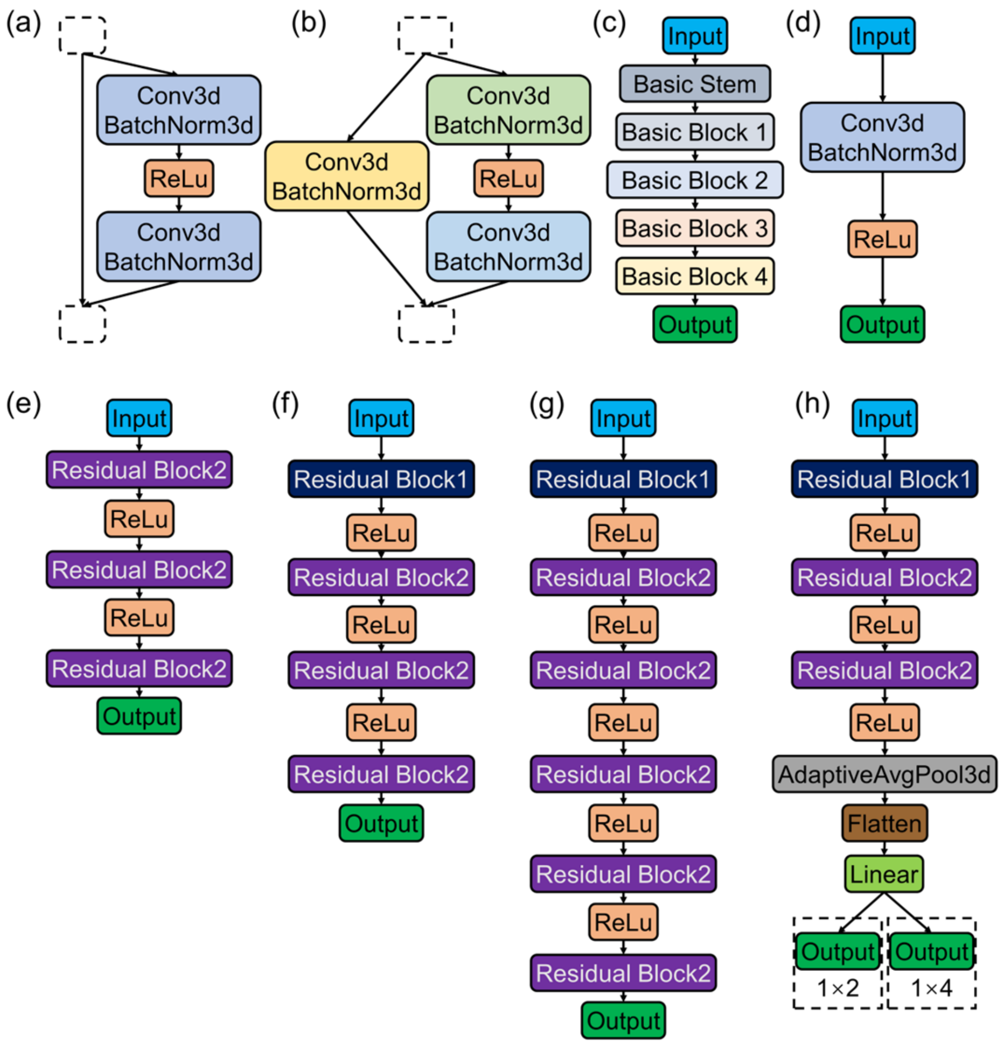

Figure S7. The detailed structure of the ResNet 50 model. Structure diagrams of (a) Residual Block 1, (b) Residual Block 2, (c) 3D ResNet 50 Model, (d) Basic Stem, (e) Basic Block 1, (f) Basic Block 2, (g) Basic Block 3, and (h) Basic Block 4. The 3D ResNet 50 model consists of a Basic Stem and four Basic Blocks, each constructed by sequentially connecting Residual Block 1, Residual Block 2, and the ReLU activation function layer by layer. The model ultimately produces the classification result.

The structures of the Basic Stem and each Basic Block are shown in Figures S7d- S7h. In the Basic Stem, the data dimensions are expanded from 8 to 64 using basic 3D convolution layers, as shown

in Figure S7d. In Basic Block 1, the first residual calculation uses identity mapping to construct an identity residual block, as shown in Figure S7e.[15] As data is processed in Basic Block 2 and Basic Block 3, as shown in Figures S7f and S7g, projection shortcuts are added during residual calculation to construct convolutional residual blocks, allowing the data to expand from 64 to 128 and 256 dimensions.[16, 17, 18, 19] In this way, the network can effectively maintain the information flow and avoid the risk of gradient vanishing.[20] In Basic Block 4, the data pass through the basic residual block and ReLU activation function multiple times and undergo processing through an average pooling layer, a flattened layer, and a fully connected layer for producing the final classification results, as shown in Figure S7h. When compared with the original ResNet 50, our model prioritizes efficiency for inputs with high pixel counts and computationally demanding 3D convolutional layers. We maintain the dimension at 64 in Basic Block 1 without increasing to 256. Additionally, in Basic Blocks 2, 3, and 4, we double the dimension just once, resulting in a dimension of 512 before entering the pooling process.

6. The principle of the basic residual block in ResNet

The basic residual block of ResNet could be generally given by:[21]

$$y_l = h(x_l) + F(x_l, W_l) \tag{1}$$

$$x_{l+1} = f(y_l) \tag{2}$$

Where x_l and x_{l+1} represent the input and output of the l block, please note that each block typically contains a multi-layer structure. W_l is a set of weights associated with the l residual unit, and could be generally given by:

$$\mathcal{W}_l = \left\{ W_{l,k} \big|_{1 \le k \le K} \right\} \tag{3}$$

K in Eqn. (3) is the number of layers in the block. The F is a residual function that represents the learned residual. If we set the function h as an identity mapping, then $h(x_l) = x_l$. The f represent the activation function. Since ReLu activation function only contains a nonlinear threshold, it can effectively solve the problem of gradient disappearance and improve the model's generalization ability. Therefore, we also select ReLu as activation function. If f is also an identity mapping, that is $x_{l+1} \equiv y_l$, we can substitute Eqn. (2) into Eqn. (1) and obtain:

$$x_{l+1} = x_l + F(x_l, W_l) \tag{4}$$

Based on the Eqn. (4), we employed an accumulation method to obtain learned features from the shallow l layer to the deep L layers, as follows:

$$x_L = x_l + \sum_{i=l}^{L-1} F(x_i, W_i) \tag{5}$$

Eqn. (5) exhibits an excellent property: The feature x_L of any deeper unit L can be expressed as the sum of a shallower unit l and a residual function F. This indicates that the model follows a residual fashion between any units L and l. Additionally, from the chain rule of backpropagation, the gradients of the reverse process can be obtained:[22]

$$\frac{\partial \text{ loss}}{\partial x_l} = \frac{\partial \text{ loss}}{\partial x_L} \cdot \frac{\partial x_L}{\partial x_l} = \frac{\partial \text{ loss}}{\partial x_L} \cdot \left(1 + \frac{\partial}{\partial x_l} \sum_{i=l}^{L-1} F(x_i, W_i)\right) \tag{6}$$

The model divides the parameters into two additive terms: items that are passed through weight layers, and items that are not. The first factor of the formula $\frac{\partial \text{ loss}}{\partial x_L}$ represents the gradient reached by the loss function at L, and ensures that information is propagated directly back to any shallower unit l. This design scheme allows any unit to be transmitted forward or backward to another unit and ensures that the model does not lose data during information transfer.

7. Comparison of training and validation results of different models

We have selected different models for comparison. DenseNet is a deep convolutional neural network architecture known for its dense connections, effectively promoting feature reuse and mitigating gradient vanishing.[23, 24] MobileNet employs depth-separable convolutions to reduce computational load and model parameters while maintaining commendable performance.[25, 26] The ShuffleNet introduces channel rearrangement operations to curtail computational complexity while preserving performance levels. Each model boasts unique design concepts and features.[27, 28]

We utilized 8 preprocessed images generated through image augmentation for a single model sample as input. The results are depicted in Figure S8. Among these models, the ResNet 50 model shows the best performance.

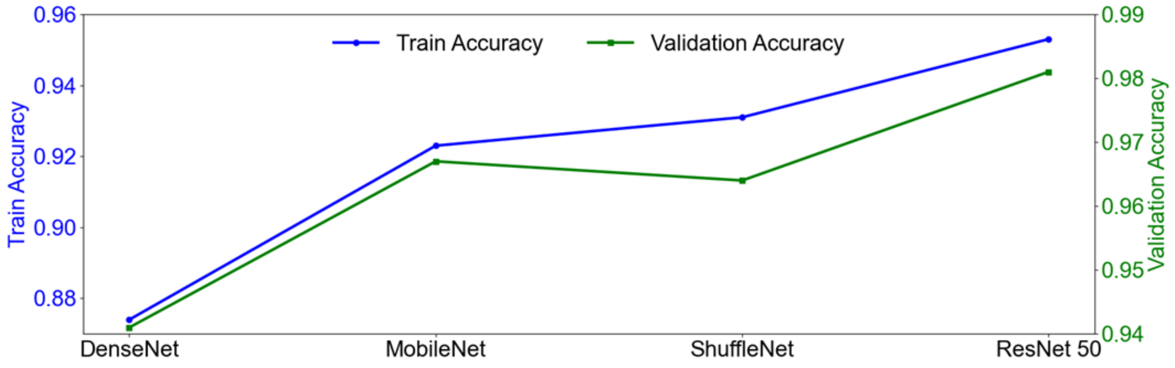

Figure S8. Comparison of training and validation results of different models. Source data are provided as a Source Data file.

8. Model development and training results

We trained an encoder-decoder architecture network based on ResNet 50 to assess the effectiveness of ResNet 50 in feature extraction from the samples. The Train Loss and Valid Loss curves in Figure S9 initially fluctuated but subsequently accurately identified the convergence

direction, resulting in a steady decrease. After 100 epochs, the loss reached an impressively low value of 0.0041746, indicating the model's ability to extract temporal RHEED features effectively.

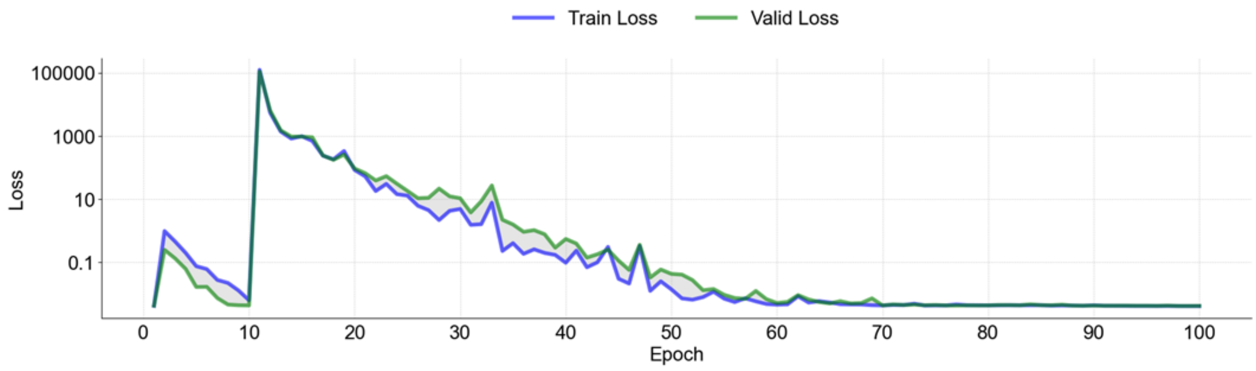

Figure S9. Training results of encoder decoder architecture model based on 3D ResNet 50. Train: training. Valid: validation. Loss: loss. The Loss curves are derived from results during model training. Source data are provided as a Source Data file.

Additionally, we tried to train a single model to predict all five classification results, including one label determining the QDs formation and four density labels, to improve efficiency. As shown in Figure S10, the Acc curve exhibits an overall upward trend, while the Loss curve shows a downward trend. Due to the more significant changes in loss values compared to accuracy values, additional simple polynomial fitting and the first derivative of the Loss curve were performed to obtain Trend and Change curves. It can be observed that the Train Trend and Train Change show a stable trend close to zero throughout the entire training process. Furthermore, the change in Valid Loss can better reflect the model's performance compared to Train Loss. The Valid Trend and Valid Change curves undergo fluctuations before gradually stabilizing, indicating that the model is progressively learning information from initial states and achieving stable convergence, resulting in a minor deviation between the model output and the dataset. However, achieving higher accuracy was challenging, as it plateaued at around 96.3% even after 100 epochs. Analyzing

data with misclassifications posed further challenges in pinpointing sources of these errors. It is not easy to find a unified pattern for these misclassified data in the dataset to optimize. Consequently, we trained two models for judging the QDs formation and classifying density, which are called the “QDs model” and the “density model”.

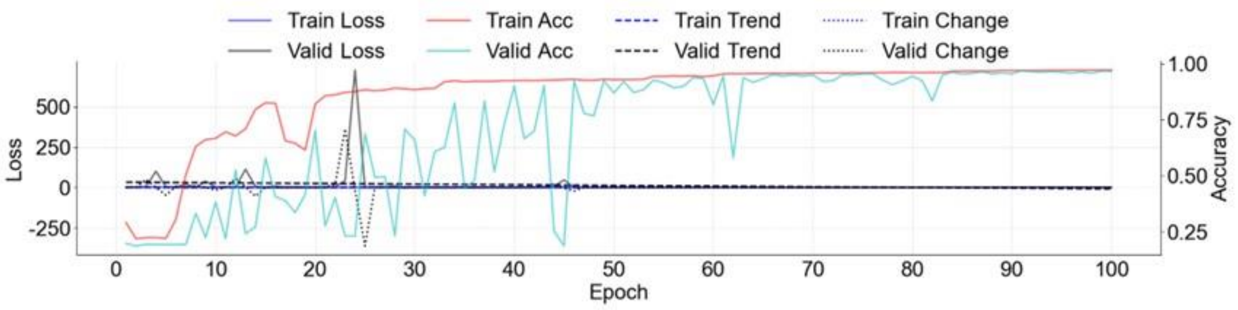

Figure S10. The training and validation data for the 3D ResNet 50 model consist of five categories: one to determine whether QDs have formed and four for density labels. Train: training. Valid: validation. Acc: accuracy. Loss: loss. The Loss and Acc curves are derived from results during model training. The Trend and Change curves are simple polynomial fit and the first derivative of the Loss curve, respectively. Source data are provided as a Source Data file.

9. Hardware wiring scheme for model deployment

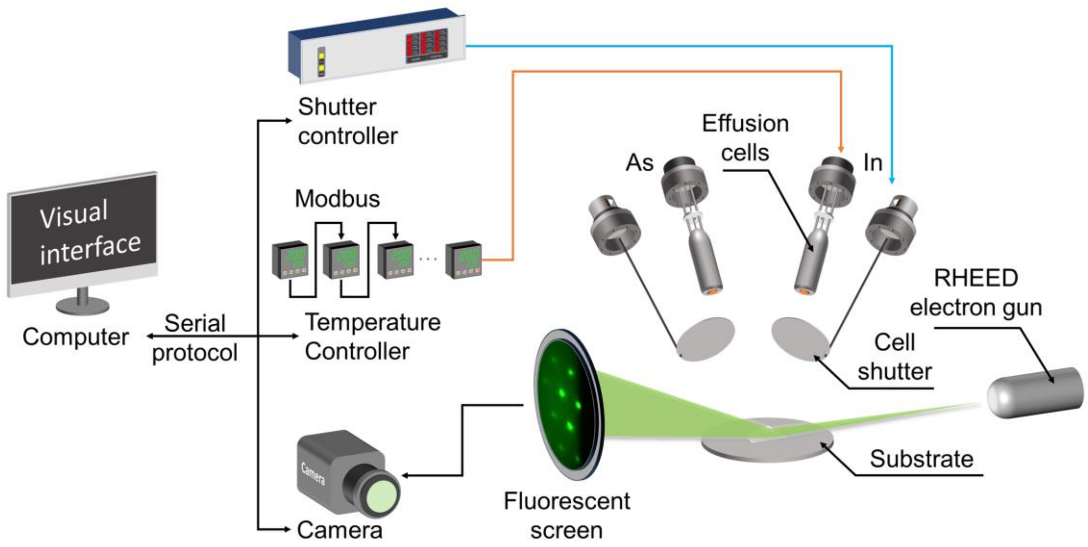

Figure S11. Hardware wiring schematic for model deployment. The molecular beam epitaxy (MBE) is controlled by the computer through a serial port, which is connected to the temperature controller, shutter controller, and camera.

10. Program interface and deployment environment

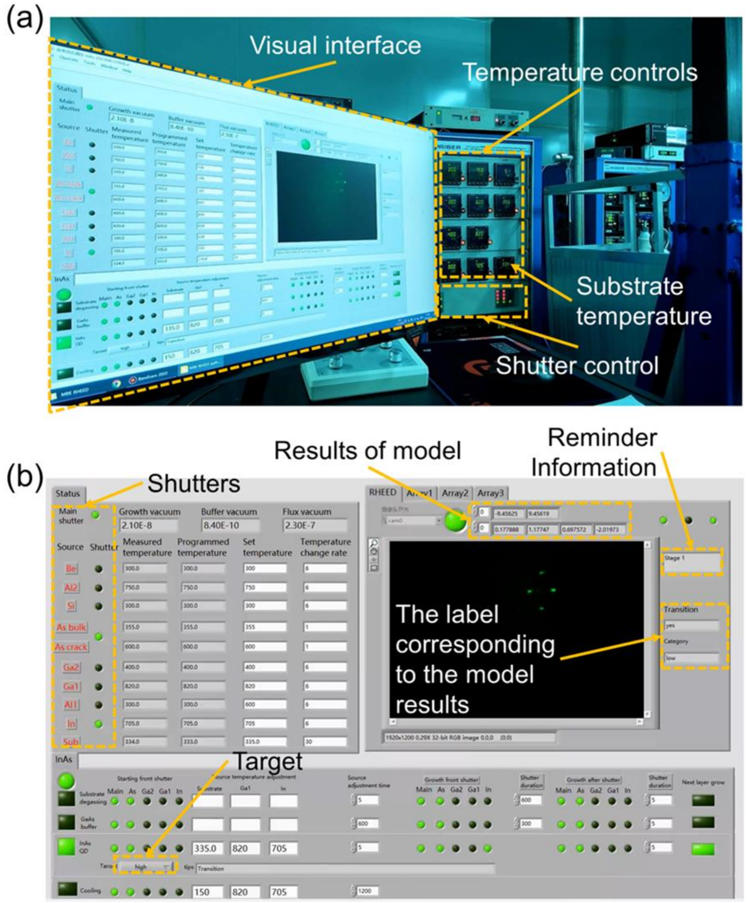

Figure S12. Program interface and deployment environment. Images of (a) the deployment environment and (b) the program interface. The visual interface, temperature controls, substrate temperature, and shutter control are clearly visible in the deployment environment. The program interface also displays shutter status and reflection high-energy electron diffraction (RHEED) video. Additionally, users can easily access reminder information, preset growth targets, and model results through the interface.

11. The QDs and density models with different wobble characteristics

Our model also exhibits potential for application in MBE systems with different wobble characteristics. The mechanical vibration is a specific manifestation of substrate wobbling, which we can simulate during data preprocessing. Using image augmentation techniques based on color curves and pixel scaling using existing data, we conducted simulations and generated data with different wobble patterns in both horizontal and vertical directions. In addition, we also perform image cropping. The offset of the image cropping selection from the edge is crucial in simulating mechanical vibration. To achieve this, we randomly generate a sine function with a period matching the substrate rotation. The amplitude of the sine function is generated using Python's random numbers. Then, we randomly set a starting point within a single cycle and collect 24 points within the length of one cycle based on this starting point. Afterward, 8 consecutive points are selected from these points as the cropping offset in the horizontal or vertical direction. Each cropped image is then resized to 300 × 300 pixels. This process results in the creation of a new dataset consisting of 8 images. Consequently, this dataset exhibits the characteristic of substrate wobbling, which was subsequently employed for fine-tuning model parameters without a new training cycle, as shown in Figure S13. As the training of the two models progresses, the Acc curve exhibits an overall upward trend, while the Loss curve shows a downward trend, resulting in an accuracy of 98.8% of QDs model and 98.4% of density model after 5 epochs. Due to the more significant changes in loss values compared to accuracy values, additional simple polynomial fitting and the first derivative of the Loss curve were performed to obtain Trend and Change curves. It can be observed that the Trend and Change curves show a stable trend close to zero throughout the entire training process, indicating that the model is progressively learning effective information and can be quickly adjusted to handle data with different wobble characteristics.

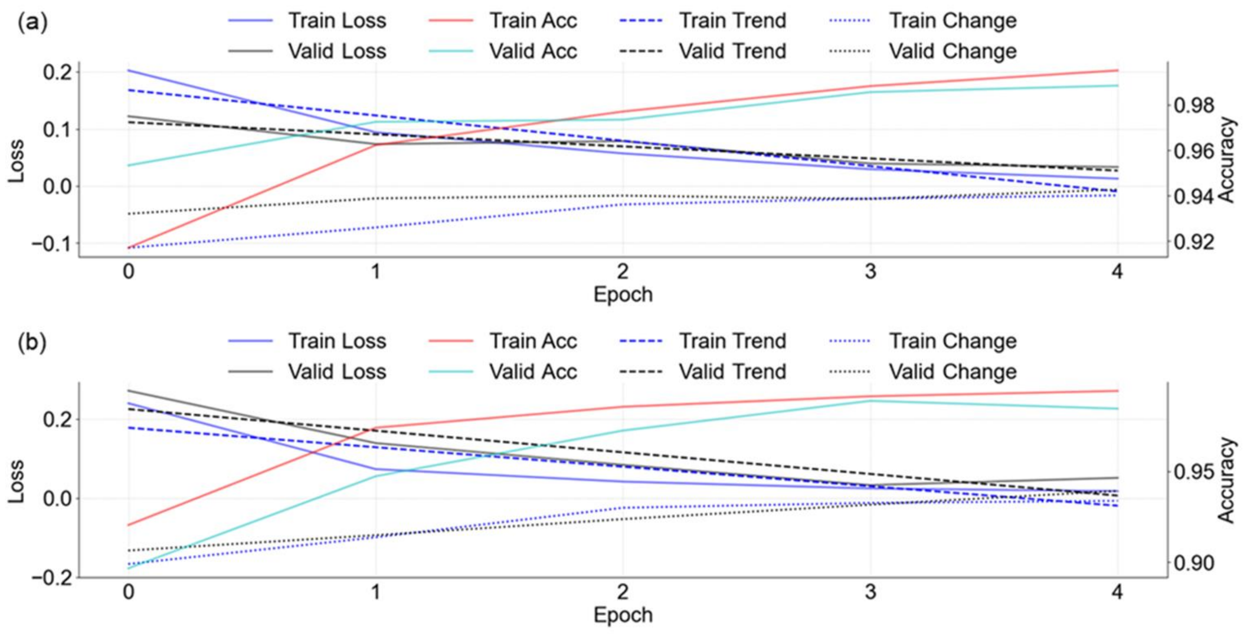

Figure S13. The QDs and density models with different wobble characteristics. Training results of fine-tuning the (a) QDs model and (b) density model on wobble characteristic data. Train: training. Valid: validation. Acc: accuracy. Loss: loss. The Loss and Acc curves are derived from results during model training. The Trend and Change curves are simple polynomial fit and the first derivative of the Loss curve, respectively. Source data are provided as a Source Data file.

12. Details of the reference sample

In this study, a sample was grown at a nominal substrate temperature of 495 °C for reference. The QD density is approximately 1.5×10^{10} cm^{-2} according to the AFM results in Figure S14a, corresponding to the “middle” label. The RHEED image was obtained after the completion of the QD growth, as shown in Figure S14b.

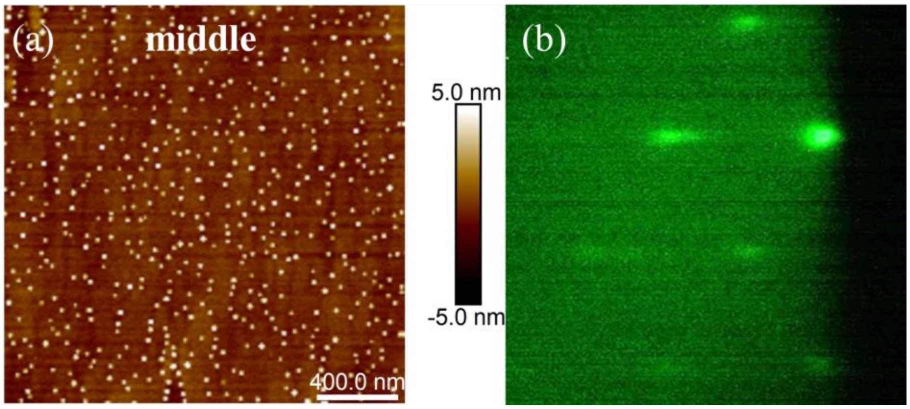

Figure S14. Details of the reference sample. (a) The 2 μm × 2 μm atomic force microscope (AFM) image of the sample, and (b) the reflection high-energy electron diffraction (RHEED) image of the sample after growth.

13. Another controlled growth experiment of low-density QDs

We also repeated the experiment under identical conditions and obtained low-density samples successfully. As shown in Figure S15a, the substrate temperature underwent a total change of 25 °C during the growth, indicating that the initial substrate temperature was unsuitable for achieving the "low" label target. Furthermore, the convergence of the blue and yellow lines at the same sequence number suggests that, upon QD formation, the "density model" promptly recognized the current RHEED sequence as consistent with the "low" label and terminated the growth process. In Figure S15b, RHEED images primarily displayed streaks during the initial growth stage, even before QD formation, as shown in Figure S15c. After QD formation, distinctive streaks and spot features promptly emerged, aligning with the characteristics of the "low" label, as depicted in Figure S15d. The density of these QDs was 3.7 × 10^8 cm^{-2}, with an average diameter of 44.8 nm and a height of 7.3 nm, as evidenced in the AFM image in Figure S15e.

Figures S15f and S15g illustrate the distribution of outputs for the QDs and density models across the entire sequence. To effectively depict data trends, we have incorporated running average plots to illustrate the model's output evolution. Before QDs formation, the model predominantly outputs "No" from the initial to around 1200th sequence, as depicted in Figure S15f. However, as the sequence approaches the blue line, the likelihood of observing "Yes" significantly rises, surpassing 55% and continuing to increase to 65% at the final sequence. It indicates the model's sensitivity in detecting the emergence of low-density QDs.

Moreover, Figure S15g presents the results of the density model. From the initial to around the 600th sequence, the cumulative probability of the model outputting "middle" and "high" labels surpasses the probability of outputting "low" labels, which indicates that the initial conditions may not be suitable for the growth of low-density QDs. From around the 600th to around the 1200th sequence, we can observe a gradual increase in the probability of the density model outputting "low". This trend continues until the final sequence, with the probability of the "low" label remaining stable at around 60%. This trend confirms that the model continuously adjusts growth parameters to create more favorable growth conditions for low-density QDs.

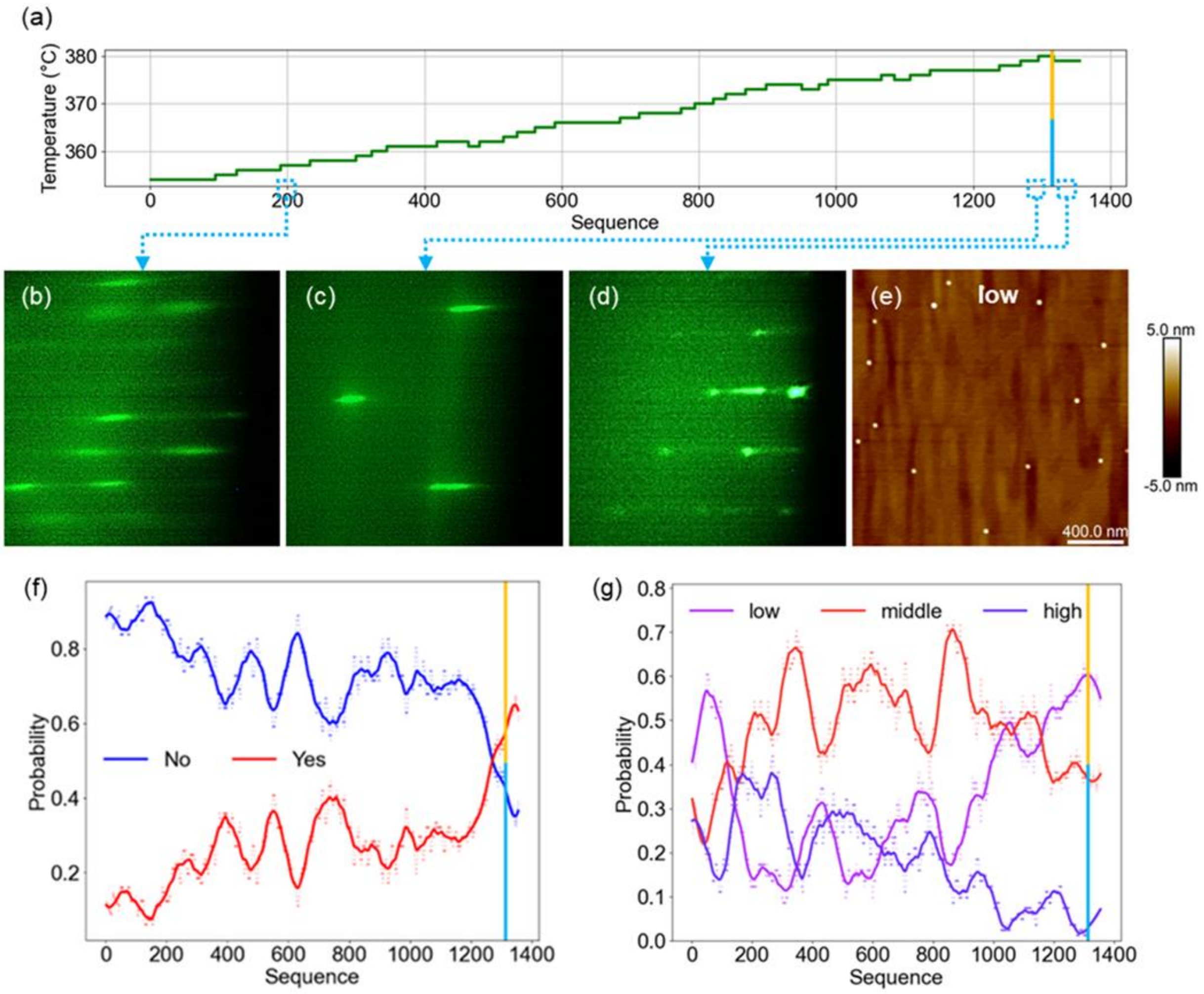

Figure S15. Additional experiment on controlling growth process of low-density quantum dots (QDs). Experiment with the "low" label as the target. Blue lines: the QD formation time. Yellow lines: the In shutter closing time. (a) Substrate temperature changes during growth. The reflection high-energy electron diffraction (RHEED) image (b) captured at 200th frame after growth; (c) before the QD formation; (d) after the QD formation; (e) the 2 μm × 2 μm atomic force microscope (AFM) image of the sample; the prediction results of (f) the "QDs model" and (g) the "density model". Dots: probability statistics of different labels based on the model results. Lines: running average plots of dots. Source data are provided as a Source Data file.

14. Controlled growth of low-density QDs with initial high-density growth conditions

We have set the initial conditions favorable for the growth of high-density QDs and conducted experiments with the "low" label as the target. As illustrated in Figure S16a, the substrate temperature underwent a total change of 33 ℃ during the growth, indicating that the initial substrate temperature was not suitable for achieving the "low" label. Furthermore, the convergence of the blue and yellow lines at the same sequence number suggests that, upon QD formation, the "density model" promptly recognized the current RHEED sequence as consistent with the "low" label and terminated the growth process. In Figure S16b, RHEED images primarily displayed streaks during the initial growth stage, even before QD formation, as shown in Figure S16c. After QD formation, distinctive streaks and spot features promptly emerged, aligning with the characteristics of the "low" label, as depicted in Figure S16d. The density of these QDs was 9.5×10^8 cm^{-2}, with an average diameter of 54.5 nm and a height of 8.3 nm, as evidenced in the AFM image in Figure S16e.

Figures S16f and S16g illustrate the distribution of outputs for the QDs model and the density model across the entire sequence. In Figure S16f, from the initial to around the 600th sequence, there is a consistently low probability at 30% of output "Yes". However, when approaching the QD formation at around the 800th sequence, a distinct trend emerges: the probability of the QDs model outputting "Yes" gradually increases to 72%.

Furthermore, in Figure S16g, due to the initial conditions favoring high-density QD growth, the probability of outputting a "low" label is significantly lower than the combined probability of "middle" and "high" labels. From the initial to the 200th sequence, the presence of the "low" label in the model's output is approximately 23%. Then, the likelihood of the model outputting the "low" label increases gradually from the 200th to the 700th sequence, surpassing 33%. Finally, the density

model consistently produces “low” outputs around the blue line, finally reaching 62%. It signifies that, under the current conditions, the material is highly inclined to foster the growth of low-density QDs.

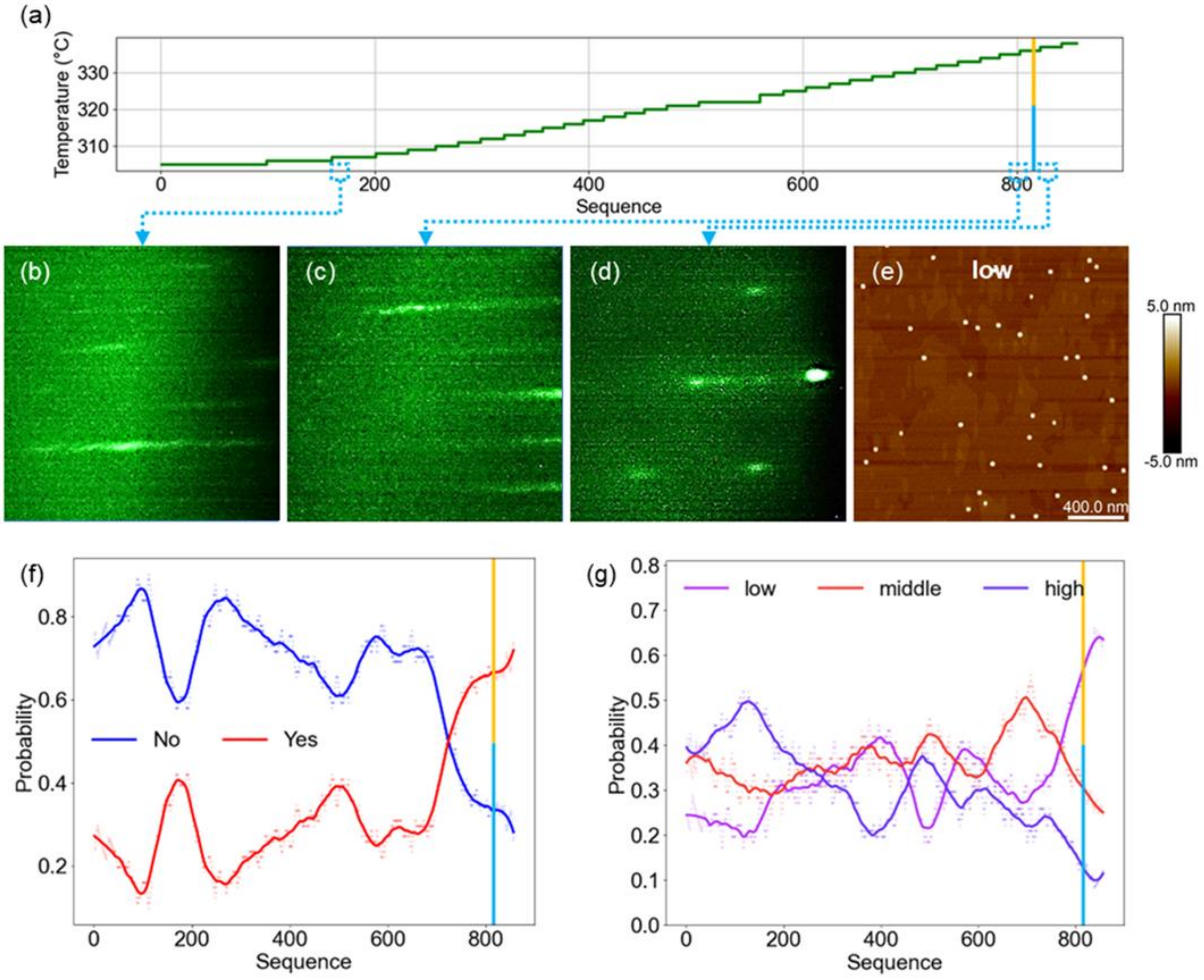

Figure S16. Controlled growth process of low-density quantum dots (QDs). Experiment with the “low” label as the target. Blue lines: the QD formation time. Yellow lines: the In shutter closing time. (a) Substrate temperature during growth. The reflection high-energy electron diffraction (RHEED) image (b) captured at 200th frame after growth; (c) before the QD formation; (d) after the QD formation; (e) the 2 μm × 2 μm atomic force microscope (AFM) image of the sample; the prediction results of (f) the “QDs model” and (g) the “density model”. Dots: probability statistics

of different labels based on the model results. Lines: running average plots of dots. Source data are provided as a Source Data file.

References

1. Alzoubi, T., Usman, M., Benyoucef, M., Reithmaier, J. P. Growth of InAs quantum dots and dashes on silicon substrates: Formation and characterization. *Journal of Crystal Growth* **323**, 422-425 (2011).

2. Steimetz, E., *et al.* In situ monitoring of InAs-on-GaAs quantum dot formation in MOVPE by reflectance-anisotropy-spectroscopy and ellipsometry. *Applied Surface Science* **107**, 203-211 (1996).

3. García, J. M., Silveira, J. P., Briones, F. Strain relaxation and segregation effects during self-assembled InAs quantum dots formation on GaAs(001). *Applied Physics Letters* **77**, 409-411 (2000).

4. Luo, X. D., Xu, Z. Y., Wang, Y. Q., Wang, W. X., Wang, J. N., Ge, W. K. Abnormal effect of growth interruption on GaSb quantum dots formation grown by molecular beam epitaxy. *Journal of Crystal Growth* **247**, 99-104 (2003).

5. Sfaxi, L., Bouzaiene, L., Sghaier, H., Maaref, H. Effect of growth temperature on InAs wetting layer grown on (113)A GaAs by molecular beam epitaxy. *Journal of Crystal Growth* **293**, 330-334 (2006).

6. Vasudevan, R. K., Tselev, A., Baddorf, A. P., Kalinin, S. V. Big-data reflection high energy electron diffraction analysis for understanding epitaxial film growth processes. *ACS Nano* **8**, 10899-10908 (2014).

7. Kwoen, J., Arakawa, Y. Classification of in situ reflection high energy electron diffraction images by principal component analysis. *Japanese Journal of Applied Physics* **60**, SBBK03 (2021).

8. Qassim, H., Feinzimer, D., Verma, A. Residual squeeze vgg16. Preprint at https://arxiv.org/abs/1705.03004.

9. Li, B., He, Y. An Improved ResNet Based on the Adjustable Shortcut Connections. *IEEE Access* **6**, 18967-18974 (2018).

10. He, K., Zhang, X., Ren, S., Sun, J. Deep residual learning for image recognition. In: *Proceedings of the IEEE conference on computer vision and pattern recognition*) (2016).

11. He, T., Zhang, Z., Zhang, H., Zhang, Z., Xie, J., Li, M. Bag of tricks for image classification with convolutional neural networks. In: *Proceedings of the IEEE/CVF conference on computer vision and pattern recognition*) (2019).

12. Aziz, L., Haji Salam, M. S. B., Sheikh, U. U., Ayub, S. Exploring Deep Learning-Based Architecture, Strategies, Applications and Current Trends in Generic Object Detection: A Comprehensive Review. *IEEE Access* **8**, 170461-170495 (2020).

13. Wu, X., Ji, Q. TBRNet: Two-Stream BiLSTM Residual Network for Video Action Recognition. *Algorithms* **13**, 169 (2020).

14. Khanna, A., Londhe, N. D., Gupta, S., Semwal, A. A deep Residual U-Net convolutional neural network for automated lung segmentation in computed tomography images. *Biocybernetics and Biomedical Engineering* **40**, 1314-1327 (2020).

15. Liu, W., Wu, G., Ren, F., Kang, X. DFF-ResNet: An insect pest recognition model based on residual networks. *Big Data Mining and Analytics* **3**, 300-310 (2020).

16. Fakhry, A., Zeng, T., Ji, S. Residual Deconvolutional Networks for Brain Electron Microscopy Image Segmentation. *IEEE Trans Med Imaging* **36**, 447-456 (2017).

17. Ganokratanaa, T., Aramvith, S., Sebe, N. Video anomaly detection using deep residual-spatiotemporal translation network. *Pattern Recognition Letters* **155**, 143-150 (2022).

18. Zhang, K., Sun, M., Han, T. X., Yuan, X., Guo, L., Liu, T. Residual Networks of Residual Networks: Multilevel Residual Networks. *IEEE Transactions on Circuits and Systems for Video Technology* **28**, 1303-1314 (2018).

19. Yu, H., Liu, J., Chen, C., Heidari, A. A., Zhang, Q., Chen, H. Optimized deep residual network system for diagnosing tomato pests. *Computers and Electronics in Agriculture* **195**, 106805 (2022).

20. Zhu, Z., *et al.* Juggler-ResNet: A Flexible and High-Speed ResNet Optimization Method for Intrusion Detection System in Software-Defined Industrial Networks. *IEEE Transactions on Industrial Informatics* **18**, 4224-4233 (2022).

21. He, K., Zhang, X., Ren, S., Sun, J. Identity Mappings in Deep Residual Networks. In: *Computer Vision – ECCV 2016* (eds Leibe B, Matas J, Sebe N, Welling M). Springer International Publishing (2016).

22. LeCun, Y., *et al.* Backpropagation Applied to Handwritten Zip Code Recognition. *Neural Computation* **1**, 541-551 (1989).

23. Zhu, Y., Shawn, N. DenseNet for dense flow. In: *2017 IEEE International Conference on Image Processing (ICIP)*) (2017).

24. Iandola, F., Moskewicz, M., Karayev, S., Girshick, R., Darrell, T., Keutzer, K. Densenet: Implementing efficient convnet descriptor pyramids. Preprint at https://arxiv.org/abs/1404.1869.

25. Sinha, D., Mohamed, E.-S. Thin MobileNet: An Enhanced MobileNet Architecture. In: *2019 IEEE 10th Annual Ubiquitous Computing, Electronics & Mobile Communication Conference (UEMCON)*) (2019).

26. Qin, Z., Zhang, Z., Chen, X., Wang, C., Peng, Y. Fd-Mobilenet: Improved Mobilenet with a Fast Downsampling Strategy. In: *2018 25th IEEE International Conference on Image Processing (ICIP)*) (2018).

27. Zhang, X., Zhou, X., Lin, M., Sun, J. Shufflenet: An extremely efficient convolutional neural network for mobile devices. In: *Proceedings of the IEEE conference on computer vision and pattern recognition*) (2018).

28. Ma, N., Zhang, X., Zheng, H.-T., Sun, J. Shufflenet v2: Practical guidelines for efficient cnn architecture design. In: *Proceedings of the European conference on computer vision (ECCV)*) (2018).
