# Supplementary material for: Machine-learning-assisted and real-time-feedback-controlled growth of InAs/GaAs quantum dots
Source: Nat Commun. 2024 Mar 29;15:2724. doi: 10.1038/s41467-024-47087-w (PMC10980817; doi:10.1038/s41467-024-47087-w)
Supplement: Supplementary file 3 — Description of Additional Supplementary Files [file 41467_2024_47087_MOESM3_ESM.docx]

File Name: Supplementary Video 1

Description: Controlled growth process of low-density QDs. Experiment with the “low” label as the target. In the video, we presented the laboratory environment where the experiment took place, showcasing the changes in the computer interface, shutter controller, and substrate temperature controller. A dedicated recording perspective of the software interface is shown in the upper right corner of the video.

File Name: Supplementary Video 2

Description: Controlled growth process of high-density QDs. Experiment with the “high” label as the target. In the video, we presented the laboratory environment where the experiment took place, showcasing the changes in the computer interface, shutter controller, and substrate temperature controller. A dedicated recording perspective of the software interface is shown in the upper right corner of the video.
